# Health-related quality of life and experience measures, to assess patients’ experiences of peripheral intravenous catheters: a secondary data analysis

**DOI:** 10.1186/s12955-023-02217-8

**Published:** 2024-01-02

**Authors:** Emily N. Larsen, Nicole Marsh, Claire M. Rickard, Gabor Mihala, Rachel M. Walker, Joshua Byrnes

**Affiliations:** 1 School of Nursing and Midwifery, Griffith University, Nathan, Australia; 2 Nursing and Midwifery Research Centre, Royal Brisbane and Women’s Hospital, Building 34, Corner Bowen Bridge Rd and Butterfield St, Herston, QLD 4029 Australia; 3 Patient-Centred Health Services, Menzies Health Institute Queensland, NHMRC Centre of Research Excellence in Wiser Wound Care, Griffith University, Nathan, Australia; 4 Alliance for Vascular Access Teaching and Research, Griffith University, Nathan, Australia; 5 School of Nursing, Midwifery and Social Work, The University of Queensland Centre for Clinical Research, Herston, QLD Australia; 6 Herston Infectious Diseases Institute, Metro North Health, Herston, QLD Australia; 7 Centre for Applied Health Economics, School of Medicine and Dentistry, Griffith University, Nathan, QLD Australia; 8 Division of Surgery, Princess Alexandra Hospital, Metro South Health, Brisbane, QLD Australia

**Keywords:** Peripheral venous catheter, Health related quality of life, Experience, Satisfaction, Utility, EQ5D, AHPEQS, FACIT

## Abstract

**Background:**

Peripheral intravenous catheters (PIVCs) are essential for successful administration of intravenous treatments. However, insertion failure and PIVC complications are common and negatively impact patients’ health-outcomes and experiences. We aimed to assess whether generic (not condition-specific) quality of life and experience measures were suitable for assessing outcomes and experiences of patients with PIVCs.

**Methods:**

We undertook a secondary analysis of data collected on three existing instruments within a large randomised controlled trial, conducted at two adult tertiary hospitals in Queensland, Australia. Instruments included the EuroQol Five Dimension - Five Level (EQ5D-5L), the Functional Assessment of Chronic Illness Therapy – Treatment Satisfaction – General measure (FACIT-TS-G, eight items), and the Australian Hospital Patient Experience Question Set (AHPEQS, 12 items). Responses were compared against two clinical PIVC outcomes of interest: all-cause failure and multiple insertion attempts. Classic descriptives were reported for ceiling and floor effects. Regression analyses examined validity (discrimination). Standardised response mean and effect size (ES) assessed responsiveness (EQ5D-5L, only).

**Results:**

In total, 685 participants completed the EQ5D-5L at insertion and 526 at removal. The FACIT-TS-G was completed by 264 and the AHPEQS by 262 participants. Two FACIT-TS-G items and one AHPEQS item demonstrated ceiling effect. Instruments overall demonstrated poor discrimination, however, all-cause PIVC failure was significantly associated with several *individual* items in the instruments (e.g., AHPEQS, ‘*unexpected physical and emotional harm’*). EQ5D-5L demonstrated trivial (ES < 0.20) responsiveness.

**Conclusions:**

Initial investigation of an existing health-related quality of life measure (EQ5D-5L) and two patient-reported experience measures (FACIT-TS-G; AHPEQS) suggest they are inadequate (as a summary measure) to assess outcomes and experiences for patients with PIVCs. Reliable instruments are urgently needed to inform quality improvement and benchmark standards of care.

**Supplementary Information:**

The online version contains supplementary material available at 10.1186/s12955-023-02217-8.

## Background

Peripheral intravenous catheters (PIVCs) are the most commonly inserted invasive device in modern healthcare delivery [1]. These devices consist of a small plastic tube (introduced into the bloodstream by a steel needle), recommended for administration of intravenous therapies ≤ 5 days. Two in every three patients entering a tertiary institution will require at least one PIVC for the delivery of essential intravenous medications and other therapies [1]. Patients often require multiple attempts to achieve successful PIVC insertion; and PIVC failure, as a result of complications such as occlusion, dislodgement, phlebitis (inflammation), local- and bloodstream-infection, occurs in one in every three PIVCs placed [2, 3]. The sequelae of multiple repeated failed attempts at PIVC insertion, and later PIVC failure include patient reported pain and distress [4], missed medication (e.g., antibiotic) doses leading to sub-optimal treatment [5], irreversible damage to vasculature [6], and, in severe cases, morbidity and mortality [7]. While the *incidence* of clinician-identified PIVC-related harm is often reported at an individual- and institution- level [8], it is essential that clinicians and policy makers further consider the patient’s self-reported health outcomes and experiences.

Health Related Quality of Life (HRQoL) measures (including patient-reported outcome measures (PROMs)) and patient-reported experience measures (PREMs) are common tools within healthcare institutions, enabling contemporaneous identification of clinical problems [9], establish suitability of healthcare interventions, improve patient-clinician communication [10], and ensure quality and safety in healthcare [11]. These instruments can be generic or disease-specific, and require validation to establish reliability and usefulness [12]. Their use in the context of PIVCs, however, has been limited.

A recent scoping review identified that no generic (whole of treatment/person) HRQoL *or* PIVC-specific instruments were used to collect/report PIVC outcomes/experiences [13]. Several studies incorporated *individual* patient-reported items into their data collection (e.g., numerical rating scales) [13]. Overall, the core domains related to five unique patient-reported outcomes including: pain, discomfort, distress, anxiety, and fear [13]. Similarly, while several individual questions related to patient experiences existed (e.g., *How much difficulty did health staff have when trying to insert an IV cannula)*, only one purpose-built PIVC-specific PREM was found [13]. This instrument was developed in partnership with industry representatives and consumers; however, it requires further testing to establish validity, reliability, and responsiveness. Within the remaining studies, domains related to patient *experiences* included satisfaction, confidence, and understanding [13]. The scoping review demonstrated a clear need for either generic or purpose-built PROMs and PREMs for use in establishing quality of care and safety, for the insertion and care of PIVCs.

## Methods

The aim of this secondary analysis was to establish the discrimination and responsiveness of two generic PREMs (The Australian Hospital Patient Experience Question Set (AHPEQS) [14]; the Functional Assessment of Chronic Illness Therapy (Treatment Satisfaction - General) (FACIT-TS-G) [15]), and one generic HRQoL measure (EuroQol Five Dimension, Five Level (EQ5D-5L) [16]), collected as an outcome of a recent clinical trial comparing two PIVC designs (integrated, non-integrated) [17]. Prior to this study, none of the selected instruments (EQ5D-5L, FACIT-TS-G, or AHPEQS) had previously been used to assess health-related outcomes for patients with PIVCs.

### Hypotheses

1 A: Null Hypothesis (discrimination). There will be no significant difference in the *experiences* of participants observed with desirable (completion of therapy) or undesirable (device failure) outcomes using the Functional Assessment of Chronic Illness Therapy (FACIT, PREM), or the Australian Hospital Patient Experience Question Set (AHPEQS, PREM).

1B: Null Hypothesis (responsiveness): There will be no significant difference in the *quality of life outcomes* of participants observed with desirable (completion of therapy) or undesirable (device failure) outcomes using the EQ5D-5L (HRQoL).

### Data collection

A multi-site randomised controlled trial (RCT) comparing the use of integrated- and non-integrated PIVCs, the OPTIMUM Trial (Australian New Zealand Clinical Trials Registry, ACTRN12617000089336) [17], was conducted between July 2017 and December 2019. In total, 1,759 adult participants were recruited from medical, surgical, and emergency settings across three adult tertiary acute care hospitals [17]. Research Nurses prospectively recruited participants prior to PIVC insertion, subsequently collecting data on patient demographics (e.g., gender, age, underlying condition), and device details (e.g., number of insertion attempts, inserting clinician, device location). Participants were assessed daily for signs and symptoms of site complications (e.g., pain, erythema/redness). Upon PIVC removal, device outcome data (e.g., reason for removal, signs, and symptoms of site complications) and patient outcome data (e.g., treatment received, PIVC replacement) were collected.

Concurrently, a convenience sample of patients (*n* = 685) were approached across two recruiting sites to provide responses to a HRQoL survey, (EQ5D-5L) and one of two patient experience surveys (FACIT-TS-G and AHPEQS). Sampling occurred Monday to Friday, based on availability of the Research Nurse. Participants were invited to participate if they were able to provide verbal informed consent and were expected to require the PIVC for > 48 h. The EQ5D-5L was administered at baseline (prior to or immediately following PIVC insertion) and at 36 to 60 h following PIVC insertion (reliant upon participant availability). The FACIT-TS-G (available for collection between July 2017 and December 2018) and AHPEQS (available between January 2019 and December 2019) were also administered at 36 to 60 h following PIVC insertion. The follow-up time-point (i.e., 36 to 60 h) was selected *a-priori*, based on the expected mean dwell time of PIVCs (local average dwell time between 1.5 and 2.5 days) [3, 18] to ensure a higher response rate (minimising attrition related to patients discharged immediately following PIVC removal). Notably, while two instruments (EQ5D-5L and FACIT-TS-G) were administered with an introductory statement asking the patient to relate responses to their outcome and experiences associated with their PIVC, one (AHPEQS) was not. This tool was instead administered with respect to the patient’s (whole) hospital experience.

### Instruments

Henceforth, all individual questions within the instruments will be referred to as ‘items’ and values recorded from responses on item scales will be ‘scores.’

#### Equation 5D-5 L

HRQoL was assessed using the EQ5D-5L [16]; this measure was selected for use in the multi-site RCT through investigator consensus, based on the widespread use of the tool and selection for use in other venous access device trials. First published in 1991 as a three-level generic measure (later adopted to a five-level option in 2009), the EQ5D is one of the most widely used quality of life instruments worldwide [19]. It has been validated in many clinical contexts (e.g., orthopaedic, cardiac settings) [20, 21], and is available in more than 150 languages [16]. The EQ5D-5L consists of five domains (mobility, self-care, usual activities, pain/discomfort, and anxiety/depression) measured against 5-levels (ranging from no problems to extreme problems) with a supplementary visual analogue scale for a self-reported health status measure (0 to 100; worst to best health). The instrument is intended for a patient population of ≥ 16 years of age and takes only a few minutes to complete [16]. EQ5D-5L responses are scored to determine a ‘summary index value’ (continuous variable, henceforth ‘utility’) as per the Australian EQ5D-5L algorithm, which accounts for up to 243 different health states (1.0, perfect health to -0.217, worse than death) [22, 23]. A disutility index value was also created by subtracting the utility estimate from one (perfect health). The introduction statement is available in Supplementary File 1.

#### FACIT-TS-G

The Functional Assessment of Chronic Illness Therapy (FACIT) measurement questionnaires, established by FACIT.org, are a series of measurements, established in over 80 languages, to assess HRQoL for a number of specific (e.g., cancer/treatment-specific) and general conditions [15]. No PIVC-specific FACIT measurement currently exists. The Functional Assessment of Chronic Illness Therapy – Treatment Satisfaction – General measure (FACIT-TS-G) was selected as the most appropriate generic PREM instrument to pilot-test in this context, intended for a population of ≥ 18 years or age undergoing treatment for chronic illness. This tool was selected by consensus of the investigator team, based on appropriateness of the included items. While the multi-centre RCT included general medical and surgical in-patients, this experience measure (designed for patients with chronic illness) was selected based on known patient demographics at the participating hospitals (which demonstrated high rates of re-admissions and underlying multi-morbidity).The tool is comprised of eight unique items which can be collated for a single summary score and is estimated to require 5 min for completion [15]. The introduction statement is available in Supplementary File 1.

#### AHPEQS

Following the rigorous development and subsequent release of the AHPEQS, developed by the Australian Commission on Safety and Quality in Health Care in 2017, use of the FACIT-TS-G was ceased, and replaced, following investigator consensus. The AHPEQS is a PREM instrument consisting of ten core items (and two sub-items) intended for use by hospitals and other healthcare providers to survey patients on their recent experiences of treatment/care [14]. The instrument is designed for a population of ≥ 18 years of age and takes approximately 10 min to complete. The introduction statement is available in Supplementary File 1.

### Outcomes of interest

The performance of the three unique instruments were assessed against two key outcomes of interest, collected during the conduct of the large multi-centre RCT. These included:

1. All-cause PIVC failure: binary variable, a composite measure of failure resulting from the most commonly occurring PIVC complications, including occlusion (the inability to infuse IV medications/fluids) [17], infiltration (movement of intravenous fluid/medication outside of the vein into the patient’s cell tissue), cell damage from an irritant infusate (extravasation) [17], phlebitis defined as clinician-reported phlebitis; patient-reported pain/tenderness (≥ 2 on a 0–10 scale) resulting in PIVC removal, *or* two or more of pain/tenderness (≥ 1 on a 0–10 scale), erythema (redness), swelling, palpable cord, vein streak, or purulent drainage) [17] (up to 24 h prior to PIVC removal), dislodgement [17], and local/bloodstream infection (according to the Centers for Disease Control/National Health and Safety Network definitions) [24].
2. Multiple insertion attempt: binary variable, defined as a PIVC requiring more than a single attempt (needle to skin) for successful insertion [17].

### Data analysis

Analysis methods were informed by previous studies, which similarly analysed generic HRQoL measures in various clinical contexts [25, 26]. Data were imported into Stata (StataCorp, Release 13. College Station, TX: StataCorp LLC) to analyse the three unique instruments’ discrimination, responsiveness, and ceiling/floor effects. *P*-values less than 0.05 were considered statistically significant. No formal corrections for multiple comparisons were applied. No data were imputed; where missing data exists, altered sample sizes are provided.

### Discrimination

(used to measure construct validity), is defined as the ability for the instruments to accurately discriminate between clinical severity levels [25] (in this case, the relationship between patient-reported scores; and PIVC failure- and non-failure events (e.g., multiple insertion attempts versus single attempt). This was analysed using generalised linear regression (gamma) model (Eq. 5D-5 L disutility scores only), regression model (ordinary least squares), ordered logistic regression model, or multinomial (polytomous) logistic regression model [27]. All regression models were multivariable, adjusting for clinically important patient/PIVC characteristics (hospital, age, gender, medical/surgical admission, PIVC type (integrated or non-integrated), device location, and gauge size).

#### Responsiveness

Three statistics were used to assess responsiveness (the absolute value of change over time, in direction and magnitude) [28] of EQ5D-5L (only); this included (i) ES (calculated as the mean EQ5D-5L score change (D) divided by standard deviation (SD) at baseline), (ii) standardised response mean (SRM) (calculated as D divided by SD of score changes), and (iii) the responsiveness statistic (calculated as D divided by SD of the constant (unchanged responses, stable participant) D) [29]. Responsiveness ES was compared against standard thresholds (< 0.2, ‘trivial’; ≥0.2 but < 0.50, ‘small’; ≥0.5 but < 0.80, ‘moderate;’ ≥0.8, ‘large’) [30].

#### Ceiling and floor effects

Assessed for all instruments (EQ5D-5L, FACIT-TS-G, AHPEQS), these were measured to test whether the instruments had the ability to represent the construct being assessed by preventing the identification of a possible genuine difference [31]. Established *a-priori*, we determined there to be a ceiling effect when ≥ 80% of responses selected the highest score of the item and a floor effect when ≥ 80% of responses select the lowest score of the item.

## Results

Of the 685 participants who completed the EQ5D-5L at baseline, 526 (77%) completed a follow-up EQ5D-5L at PIVC removal. The FACIT-TS-G was provided as a supplementary instrument to 264 participants (50%), with the remining 262 participants completing the AHPEQS instrument. Most participants were male (67%), with a mean age of 62 (SD 15.7) years (Table 1). A large majority of participants (95%) were admitted for emergent- or planned- surgery, and were from a single large tertiary hospital (94%); this was representative of the patients in the larger multi-centre RCT sample [17]. Devices were commonly 22 or 24 gauge/size (76%), inserted in the forearm (71%). Patient and device characteristics were similar between the FACIT-TS-G and AHPEQS groups; there were no AHPEQS instruments completed at site two (small tertiary hospital). Overall, 20% (103/524) of participants completing follow-up instruments had experienced two or more PIVC insertion attempts; 30% (155/524) experienced all-cause PIVC failure, with phlebitis the most reported complication (*n* = 73, 14%).

**Table 1:** Participant and device characteristics

	EQ5D-5L T0 (***N***=685)	EQ5D-5L T1 (***N***=526)	FACIT (***N***=264)	AHPEQS (***N***=262)
PARTICIPANT CHARACTERISTICS	N(%)^**a**^	N(%)^**a**^	N(%)^**a**^	N(%)^**a**^
Age (in years) (mean, SD) *Range 18-99*	61, 16.1	62, 15.7	61.8, 16.2	62, 15.2
Hospital				
Site 1	607 (89)	497 (94)	235 (89)	262 (100)
Site 2	78 (11)	29 (6)	29 (11)	0 (0)
Gender (male)	454 (66)	354 (67)	174 (66)	181 (69)
Reason for admission				
Surgical	623 (91)	501 (95)	242 (92)	259 (99)
Medical	62 (9)	25 (5)	22 (8)	3 (1)
Multiple insertion attempts (yes)	145 (21)	(*n*=524) 103 (20)	(*n*=263) 53 (20)	(*n*=261) 49 (19)
Two	105 (72)	76 (74)	35 (66)	40 (82)
Three or more	40 (28)	27 (26)	18 (34)	9 (18)
Ultrasound used (yes)	10 (1)	7 (1)	7 (1)	0 (0)
**DEVICE CHARACTERISTICS**	***N*** **=679**	***N*** **=526**	***N*** **=264**	***N*** **=262**
PIVC type inserted				
Integrated	323 (48)	247 (47)	123 (47)	124 (47)
Non-integrated	356 (52)	279 (53)	141 (53)	138 (53)
Gauge size				
22/24 gauge	495 (73)	399 (76)	203 (77)	195 (74)
20 gauge	176 (26)	121 (23)	55 (21)	67 (26)
16/18 gauge	8 (1)	6 (1)	6 (2)	0 (0)
Device location				
Forearm	464 (68)	371 (71)	167 (63)	204 (78)
Hand/Wrist	115 (17)	83 (16)	51 (19)	32 (12)
Antecubital	82 (12)	61 (12)	38 (14)	23 (9)
Other	18 (3)	11 (2)	8 (3)	3 (1)
**OUTCOMES**				
Dwell time (mean, SD)	2.7, 1.6	3.1, 1.5	3.0, 1.4	3.2, 1.5
Insertion Pain (NRS 0-10) (mean, SD)	2.6, 2.3	2.6, 2.3	2.7, 2.3	2.6, 2.3
All-cause failure	196 (29)	155 (30)	87 (33)	68 (26)
Complications:^b^				
Infiltration	46 (7)	40 (8)	17 (6)	23 (9)
Occlusion	22 (3)	18 (3)	12 (5)	6 (2)
Phlebitis	101 (15)	73 (14)	45 (17)	28 (11)
Dislodgement/leaking	60 (9)	50 (10)	27 (10)	23 (1)
Unknown	3 (<1)	0 (0)	2 (<1)	0 (0)
Serious adverse event				
Bloodstream infection^c^	5 (1)	5 (1)	1 (<1)	4 (2)

^a^Unless otherwise noted; ^b^more than one option possible; EuroQol Five Dimension, Five Level = EQ5D-5L; T0 = baseline timepoint; T1 = follow-up timepoint; Australian Hospital Patient Experience Question Set = AHPEQS; Functional Assessment of Chronic Illssness Therapy – Treatment Satisfaction – General measure = FACIT-TS-G; PIVC = Peripheral Intravenous sCatheter; *SD* Standard deviation, *NRS* Numerical rating scale; ^c^unrelated to the PIVC

 At baseline, more than half of participants reported (in relation to their PIVC) either ‘no’ or ‘slight’ problems on the EQ5D-5L, with mobility, personal-care, pain/discomfort, or anxiety/depression (68%, 74%, 51%, and 79%, respectively), whilst slightly less than half reported ‘no’ or ‘slight’ problems for usual activities (49%) (Table 2). This was consistent at follow up with more than half of participants reporting either no or slight problems with mobility, personal-care, pain/discomfort, or anxiety/depression (67%, 73%, 61%, and 83%, respectively), and 49% for usual activities. The self-reported overall health score was 61.4 (SD 22.9) and 64.7 (SD 21.3) at baseline and follow-up, respectively. The mean EQ5D-5L utility score was 0.52 (95% confidence interval [CI] 0.49,0.55) and 0.55 (95% CI 0.52,0.58) at baseline and follow-up, respectively.

Participants completing the FACIT-TS-G instrument demonstrated poorer outcomes/experiences in their responses, compared to that which they reported for the EQ5D-5L. Compared to what was expected, participants rated the effectiveness and side effects (of their PIVC) as a little/a lot better in 56% and 44% of responses, respectively. Participants most frequently answered “completely agree” that they received assistance in evaluating the effects of their treatment, received treatment that were right for them, and were satisfied with the effects of treatment (49%, 66% and 57%, respectively). Among participants, 84% would recommend this treatment to others and 82% would choose it again. Overall care was reported as very good or excellent among 67% of respondents.

Responses to the AHPEQS instrument (regarding the overall hospital episode) demonstrated positive experiences, with participants responding that they ‘always’ or ‘mostly’ had their views and concerns listened to (89%), had their individual needs met (91%), felt cared for (97%), were involved in decision-making (82%), were kept informed (88%), believed their staff communications with each other (90%), received adequate pain relief (91%), and felt confident in their safety of care (97%). Overall quality of treatment was reported as ‘very good’ or ‘good’ for 96% of all respondents. Despite this, 19% of participants reported unintentional harm because of their care, including emotional distress, physical harm, or both.

**Table 2:** Responses EQ5D-5L, FACIT-TS-G, AHPEQS

Instrument	Scale					
EQ5D-5L	No	Slight	Moderate	Severe	Extreme	Score
T0 (*n*=685)					61.4 (SD 22.9)	
Mobility	343 (50)	125 (18)	80 (12)	46 (7)	91 (13)	
Personal care	364 (53)	146 (21)	79 (12)	45 (7)	51 (7)	
Usual activities	209 (31)	122 (18)	105 (15)	40 (6)	209 (31)	
Pain/discomfort	236 (34)	117 (17)	199 (29)	103 (15)	30 (4)	
Anxiety/depression	440 (64)	105 (15)	111 (16)	21 (3)	8 (1)	
T1 (*n*=526)					64.7 (SD 21.3)	
Mobility	256 (49)	97 (18)	77 (15)	35 (7)	61 (12)	
Personal care	284 (54)	100 (19)	70 (13)	36 (7)	36 (7)	
Usual activities	160 (30)	99 (19)	92 (17)	40 (8)	135 (26)	
Pain/discomfort	209 (40)	113 (21)	125 (24)	56 (11)	23 (4)	
Anxiety/depression	330 (63)	103 (20)	75 (14)	7 (1)	11 (2)	
**AHPEQS** (*n*=262)	**Always**	**Mostly**	**Sometimes**	**Rarely**	**Never**	**Didn’t apply**
Views and concerns	156 (60)	77 (29)	25 (10)	2 (1)	1 (<1)	1 (<1)
Individual needs	168 (64)	71 (27)	19 (7)	4 (2)	0 (0)	0 (0)
Staff explanation (*n*=23)	5 (22)	7 (30)	7 (30)	4 (17)	0 (0)	0 (0)
Cared for	207 (79)	48 (18)	7 (3)	0 (0)	0 (0)	0 (0)
Decision making	152 (58)	64 (24)	32 (12)	11 (4)	3 (1)	0 (0)
Informed	164 (63)	65 (25)	22 (8)	11 (4)	0 (0)	0 (0)
Inter-staff communication	196 (75)	39 (15)	23 (9)	4 (2)	0 (0)	0 (0)
Pain relief	198 (76)	40 (15)	8 (3)	2 (1)	14 (5)	0 (0)
Confidence in safety (*n*=261)	219 (84)	33 (13)	8 (3)	1 (<1)	0 (0)	0 (0)
	**Physical harm**	**Emotional distress**		**Both**	**No**	
Unexpected harm	9 (3)	22 (8)		19 (7)	212 (81)	
	**Yes**	**No**		**Not sure**	**Didn’t discuss**	
Harm discussed (*n*=50)	34 (69)	15 (30)		0 (0)	1 (<1)	
	**Very good**	**Good**	**Average**	**Poor**	**Very poor**	
Overall quality (*n*=261)	209 (80)	43 (16)	8 (3)	1 (<0)	0 (0)	
**FACIT-TS-G** (*n*=264)	**Lot worse**	**Little worse**	**About the same**		**Little better**	**Lot better**
Effectiveness	9 (3)	10 (4)	96 (36)		61 (23)	88 (33)
Side effects	8 (3)	20 (8)	120 (45)		39 (15)	77 (29)
	**No**	**Some extent**		**Most part**	**Completely**	
Doctor(s) help	23 (9)	40 (15)		71 (27)	130 (49)	
Right for you	4 (2)	16 (6)		69 (26)	175 (66)	
Satisfied	13 (5)	24 (9)		77 (29)	150 (57)	
	**No**	**Maybe**		**Yes**		
Recommend	14 (5)	28 (11)		222 (84)		
Choose again	13 (5)	35 (13)		216 (82)		
	**Poor**	**Fair**	**Good**	**Very good**	**Excellent**	
Overall rating	7 (3)	15 (6)	66 (25)	106 (40)	70 (27)	

EuroQol Five Dimension, Five Level = EQ5D-5L; T0 = baseline timepoint; T1 = follow-up timepoint; Australian Hospital Patient Experience Question Set = AHPEQS; Functional Assessment of Chronic Illness Therapy – Treatment Satisfaction – General measure = FACIT-TS-G; SD = standard deviation.

All-cause PIVC failure and multiple insertion attempts were associated with several individual items in the three instruments (E5D-5L, FACIT-TS-G, AHPEQS) (presented here as coefficients and *p*-values in text). For those with all-cause failure, participants were more likely to report increased *mobility problems* within the EQ5D-5L (utility − 0.022, p = 0.038; disutility 0.02, p = 0.04 ‘*slight problems*’ 0.802, p = 0.004; ‘*moderate problems*’ 0.642, p = 0.035; and ‘*unable to mobilise*’ 0.713 p = 0.033) (Table 3) (detailed EQ5D-5L analysis results available in Supplementary Table 1). In the ordered logistic regression only, all-cause failure significantly correlated with increased problems with ‘*usual activities*’ (0.371, p = 0.042). Multiple insertion attempts were not associated with any EQ5D-5L items.

**Table 3:** EQ5D-5L discrimination results

***EQ5D-5L (n***=526)	Reg	***P*** value	Ologit	***P*** value	Mlogit	***P*** value	Reg	***P*** value	Ologit	***P*** value	Mlogit	***P*** value
Utility score (overall)	-0.060 (-0.131,0.011)	0.099	~	~	~	~	-0.040 (-0.124, 0.043)	0.343	~	~	~	~
Disutility score (overall)	0.122 (-0.041, 0.285)	0.142	~	~	~	~	0.068 (-0.118, 0.255)	0.473	~	~	~	~
Mobility (Ref: no)	**0.325 (0.054, 0.597)**	**0.019**	0.528 (0.161,0.896)	0.005	**Slight: 0.802 (0.256,1.348) Moderate: 0.642 (0.045,1.24)** Severe: 0.518 (-0.334,1.370) **Unable: 0.713 (0.057,1.369)**	**0.004** **0.035** 0.233 **0.033**	0.211 (-0.106, 0.529)	0.192	0.275 (-0.168,0.719)	0.224	Slight: -0.137 (-0.834,0.559) Moderate: 0.308 (-0.387, 1.002) Severe: 0.001 (-0.992, 0.994) Unable: 0.566 (-0.175, 1.308)	0.699 0.385 0.999 0.134
Utility	**-0.022 (-0.043;-0.001)**	0.038	~	~	~	~	-0.014 (-0.039,-0.010)	0.259	~	~	~	~
Disutility	**0.020 (0.001, 0.039)**	**0.040**	~	~	~	~	0.013 (-0.009,0.036)	0.257	~	~	~	~
Personal care (Ref: no)	0.020 (-0.226,0.267)	0.871	0.127 (-0.25,0.504)	0.509	Slight: 0.429 (-0.085,0.943) Moderate: 0.010 (-0.616,0.637) Severe: 0.177 (-0.666,1.021) Unable: -0.072 (-0.916,0.773)	0.102 0.974 0.680 0.868	0.104 (-0.184,0.392)	0.478	0.074 (-0.375,0.523)	0.746	Slight: -0.328 (-0.991,0.335) Moderate:-0.038 (-0.753,0.678) Severe: 0.289 (-0.64,1.218) Unable: 0.293 (-0.636,1.222)	0.332 0.917 0.541 0.537
Utility	-0.002 (-0.02, 0.015)	0.799	~	~	~	~	-0.008 (-0.028, 0.012)	0.438	~	~	~	~
Disutility	0.002 (-0.014, 0.018)	0.815	~	~	~	~	0.007 (-0.012, 0.026)	0.446	~	~	~	~
Usual activities (Ref: no)	0.294 (-0.018,0.606)	0.065	**0.371 (0.014,0.727)**	**0.042**	Slight: 0.097 (-0.517,0.711) Moderate: 0.502 (-0.111,1,115) Severe: 0.278 (-0.552,1.107 Unable: 0.475 (-0.068, 1.018	0.757 0.109 0.512 0.087	0.033 (-0.332,0.398)	0.858	0.108 (-0.308,0.524)	0.611	Slight: 0.432 (-0.248,1.113) Moderate: 0.334 (-0.39,1.057) Severe: -0.088 (-1.125,0.949) Unable: 0.175 (-0.468, 0.818)	0.213 0.366 0.868 0.593
Utility	-0.019 (-0.0415,0.004)	0.103	~	~	~	~	-0.002 (-0.029,0.024)	0.863	~	~	~	~
Disutility	*NA*	*NA*	~	~	~	~	*NA*	*NA*	~	~	~	~
Pain/discomfort (Ref: no)	0.205 (-0.027,0.438)	0.084	0.29 (-0.073,0.653)	0.118	Slight: -0.475 (-1.065,0.115) Moderate: 0.245 (-0.274,0.764) Severe: 0.566 (-0.104,1.236 Extreme: 0.325 (-0.634,1.283	0.115 0.355 0.098 0.506	0.05 (-0.223,0.322)	0.720	0.095 (-0.335,0.525)	0.665	Slight: 0.137 (-0.494,0.769) Moderate: -0.080 (-0.728,0.567) Severe: 0.502 (-0.281,1.285) Extreme: -0.133 (-1.280,1.015)	0.670 0.808 0.209 0.821
Utility	-0.014 (-0.033,0.004)	0.125	~	~	~	~	-0.007 (-0.029, 0.014)	0.522	~	~	~	~
Disutility	0.013 (-0.004, 0.030)	0.128	~	~	~	~	0.006 (-0.014,0.026)	0.543	~	~	~	~
Anxiety/depression (Ref: no)	-0.008 (-0.192,0.176)	0.931	-0.088 (-0.493,0.317)	0.670	Slight: -0.359 (-0.898,0.18) Moderate: 0.748 (-0.508,0.658) Severe: 0.652 (-1.266,2.57) Extreme: -0.220 (-1.658,1.217)	0.192 0.801 0.505 0.764	0.074 (-0.141,0.289)	0.502	0.140 (-0.323,0.604)	0.553	Slight: -0.035 (-0.647,0.578) Moderate: 0.153 (-0.527,0.834) Severe: 0.354 (-1.889,2.598) Extreme: 0.247 (-1.306,1.799)	0.912 0.658 0.757 0.755
Utility	0.002 (-0.020, 0.023)	0.888	~	~	~	~	-0.009 (-0.034,0.017)	0.513	~	~	~	~
Disutility	-0.002 (-0.022,0.019)	0.880	~	~	~	~	0.008 (-0.016,0.031)	0.514	~	~	~	~

EuroQol Five Dimension, Five Level = EQ5D-5L; reg = regression analysis ; Ologit = ordered logistic regression analysis ; Mlogit = multinomial logistic regression analysis; Ref = reference; NA = Not analysable; Bold text indicates *p* value =<0.05

With respect to items in the FACIT-TS-G, all-cause PIVC failure was significantly associated with lower *effectiveness* (-0.558, p = 0.000), *satisfaction* (-0.465, p = 0.000), *likelihood to recommend PIVC to others* (-0.17, p = 0.015), *likelihood to choose PIVC again* (-0.226, p = 0.002), and *overall rating* (-0.392, p = 0.003) (Table 4) (detailed FACIT-TS-G analysis results available in Supplementary Table 2). Additionally, all-cause failure was significantly associated with a reduced likelihood of participants reporting that doctors didn’t help them (“*at all*”) e*valuate the effects of their PIVC* (i.e., decreased likelihood of reporting doctors helping them) (-1.891, p = 0.015). Participants with multiple PIVC insertion attempts were significantly more likely to report lower *satisfaction* “*to some extent*” with their PIVC (1.789, p = 0.002).

**Table 4:** FACIT-TS-G discrimination results

FACIT-TS-G (***n***=264)	Reg	***P*** value	Ologit	***P*** value	Mlogit	***P*** value	Reg	***P*** value	Ologit	***P*** value	Mlogit	***P*** value
Effectiveness (Ref: about the same)	**-0.558 (-0.835,-0.280)**	**0.000**	**-0.987 (-1.519,-0.455)**	**0.000**	**A lot worse: 2.955 (0.763,5.147)** A little worse: 1.127 (-0.347,2.601) A little better: -0.342 (-1.126,0.443) A lot better: -0.562 (-1.299,0.175)	**0.008** 0.134 0.393 0.135	-0.019 (-0.349,0.312)	0.912	-0.05 (-0.659,0.559)	0.872	A lot worse: -0.945 (-3.766,1.876) A little worse: 1.145 (-0.428,2.719) A little better: 0.156 (-0.736,1.047) A lot better: -0.04 (-0.912,0.832)	0.511 0.154 0.732 0.928
Side Effects (Ref: about the same)	-0.282 (-0.577,0.0142)	0.062	-0.461 (-0.986,0.065)	0.086	A lot worse: 1.677 (-0.053, 3.406) A little worse: 0.733 (-0.351,1.818) A little better: -0.292 (-1.202,0.617) A lot better: -0.075 (-0.765,0.615)	0.057 0.185 0.528 0.832	0.198 (-0.154,0.55)	0.269	0.270 (-0.341,0.882)	0.386	A lot worse: NA A little worse: 1.216 (-0.046,2.479) A little better: 0.425 (-0.552,1.402) A lot better: 0.543 (-0.298,1.385)	NA 0.059 0.394 0.508
Doctor(s) help (Ref: completely)	0.199 (-0.054,0.453)	0.123	0.412 (-0.13,0.954)	0.136	**Not at all: -1.891 (-3.417,-0.364)** To some extent: 0.278 (-0.564,1.12) For the most part: -0.387 (-1.108,0.335)	0.015 0.518 0.293	-0.111 (-0.413,0.190)	0.468	-0.192 (-0.833,0.449)	0.557	Not at all: 0.524 (-0.727,1.775) To some extent: 0.437 (-0.545,1.419) For the most part: -0.749 (-1.712,0.213)	0.412 0.383 0.127
Right for you (Ref: completely)	-0.067 (-0.251,0.118)	0.476	-0.096 (-0.686,0.493)	0.748	Not at all: 1.495 (-1.099,4.088) To some extent: 0.145 (-1.108,1.399) For the most part: -0.107 (-0.773, 0.559)	0.259 0.82 0.753	0.001 (-0.218,0.221)	0.991	-0.001 (-0.71,0.707)	0.997	Not at all: NA To some extent: 0.342 (-0.957,1.642) For the most part: -0.183 (-0.991,0.626	NA 0.606 0.658
Satisfied (Ref: completely)	**-0.465 (-0.69,-0.24)**	**0.000**	**-1.01 (-1.571,-0.449)**	**0.000**	**Not at all: 2.629 (1.160,4.098)** To some extent: 0.859 (-0.225,1.944) For the most part: 0.402 (-0.262,1.065)	**0.000** 0.120 0.236	-0.214 (-0.482,0.054)	0.117	-0.585 (-1.227,0.057)	0.074	Not at all: -0.860 (-3.204,1.483) **To some extent: 1.789 (0.66,2.918)** For the most part: 0.312 (-0.493,1.117)	0.472 **0.002** 0.447
Recommend (Ref: yes)	**-0.17 (-0.308,-0.033)**	**0.015**	**-0.905 (-1.671,-0.139)**	**0.021**	**No: 1.52 (0.184,2.856)** Maybe: 0.507 (-0.408,1.422)	**0.026** 0.277	-0.018 (-0.181,0.146)	0.832	-0.172 (-1.092,0.748)	0.714	No: 0.469 (-1.112,2.051) Maybe: 0.094 (-1.008,1.195)	0.561 0.868
Choose again (Ref: yes)	**-0.226 (-0.366,-0.085)**	**0.002**	**-0.928 (-1.643,-0.213)**	**0.011**	**No: 2.607 (1.084,4.131)** Maybe: 0.222 (-0.622,1.066)	**0.001** 0.606	-0.095 (-0.262,0.072)	0.262	-0.519 (-1.377,0.339)	0.236	No: 0.49 (-1.167,2.147) Maybe: 0.537 (-0.439,1.513)	0.562 0.281
Overall rating (Ref: very good)	**-0.392 (-0.648,-0.135)**	**0.003**	**-0.681 (-1.2,-0.163)**	**0.010**	Poor: NA Fair: 0.603 (-0.703,1.91) Good: 0.248 (-0.503,1.00) Excellent: -0.137 (-0.868,0.593)	NA 0.365 0.517 0.712	0.067 (-0.239,0.372)	0.667	0.062 (-0.548,0.672)	0.842	Poor: NA Fair: 0.535 (-0.9,1.97) Good: 0.379 (-0.474,1.232) Excellent: 0.4 (-0.496,1.295)	NA 0.465 0.384 0.382

Functional Assessment of Chronic Illness Therapy – Treatment Satisfaction – General measure = FACIT-TS-G; reg = regression analysis ; Ologit = ordered logistic regression analysis ; Mlogit = multinomial logistic regression analysis; Ref = reference; NA = Not analysable; Bolt text indicate *p* value =<0.05

All-cause PIVC failure was significantly associated with participants experiencing unexpected ‘*physical and emotional harm*’ (1.577, p = 0.005) in the AHPEQS (Table 5) (detailed AHPEQS analysis results available in Supplementary Table 3). Additionally, all-cause failure was significantly associated with participants reporting higher involvement in ‘*decision-making*’ (0.575, p = 0.049) and greater ‘*inter-staff communication*’ (0.213, p = 0.046). Multiple PIVC insertion attempts were not associated with any AHPEQS items.

**Table 5:** AHPEQS discrimination results

AHPEQS (*n*=261)	Reg	***P*** value	Ologit	***P*** value	Mlogit	***P*** value	Reg	***P*** value	Ologit	***P*** value	Mlogit	***P*** value
Views and concerns (Ref: always)	0.093 (-0.124,0.31)	0.401	0.22 (-0.352,0.792)	0.451	Mostly: 0.319 (-0.331,0.969) Sometimes: -0.297 (-1.509,0.916) Rarely:1.64 (-2.186,5.480) Never: NA	0.336 0.631 0.4 NA	0.049 (-0.199,0.297)	0.697	0.103 (-0.567,0.773)	0.763	Mostly: - 0.070 (-0.857, 0.717) Sometimes: 0.345 (=0.785,1.476) Rarely: 1.138 (-4.841,7.118) Never: NA	0.861 0.550 0.709 NA
Individual needs (Ref: always)	0.099 (-0.105,0.304)	0.341	0.297 (-0.299, 0.892)	0.329	Mostly: 0.241 (-0.4s32,0.915) Sometimes: 0.169 (-0.952,1.290) Rarely: 2.119 (-0.697,4.935)	0.483 0.767 0.140	-0.057 (-0.290,0.176)	0.631	-0.134 (-0.826,0.557)	0.704	Mostly: -0.030 (-0.810,0.749) Sometimes: -0.638 (-2.036,0.76 Rarely: NA	0.939 0.371 NA
Staff explanation (*n*=23)	0.171 (-1.597,1.939)	0.835	0.342 (-2.182,2.866)	0.790	NA	NA	0.198 (-1.600,1.997)	0.813	0.365 (-2.32,3.049)	0.790	NA	NA
Cared for (Ref: always)	0.095 (-0.049,0.238)	0.195	0.45 (-0.235,1.135)	0.198	Mostly: 0.399 (-0.331,1.128) Sometimes: 0.694 (-0.975,2.363)	0.284 0.415	-0.041 (-0.204,0.123)	0.626	-0.227 (-1.070,0.617)	0.598	Mostly: -0.237 (-1.136,0.663) Sometimes: -0.385 (-2.743,1.974)	0.606 0.749
Decision making (Ref: always)	0.196 (-0.08,0.472)	0.163	**0.575 (0.002,1.148)**	**0.049**	Mostly: 0.311 (-0.428,1.050) **Sometimes: 1.1 (0.191,2.009)** Rarely: 0.961 (-0.543,2.465) Never: NA	0.409 **0.018** 0.210 NA	-0.13 (-0.444,0.185)	0.418	-0.346 (-1.040,0.348)	0.329	Mostly: -0.565 (-1.497,0.366) Sometimes: 0.074 (-0.926,1.075) Rarely: -0.077 (-1.925,1.771) Never: NA	0.234 0.885 0.935 NA
Informed	0.192 (-0.048,0.431)	0.116	0.471 (-0.113,1.054)	0.114	NA	NA	-0.044 (-0.317,0.229)	0.752	-0.045 (-0.741,0.650)	0.899	NA	NA
Inter-staff comm. (Ref: always)	**0.213 (0.004,0.422)**	**0.046**	0.621, (-0.032,1.274)	0.062	Mostly: 0.480 (-0.361,1.321) Sometimes: 0.639 (-0.375,1.653) Rarely: 1.841 (-0.342,4.02)	0.263 0.217 0.098	0.093 (-0.145,0.332)	0.442	0.495 (-0.248,1.237)	0.192	**Mostly: 0.905 (0.003,1.807)** Sometimes: 0.454 (-0.76,1.668) Rarely: NA	**0.049** 0.464 NA
Pain relief (Ref: always)	-0.068 (-0.423,0.287)	0.706	0.309 (-0.342,0.96)	0.353	Mostly: 0.626 (-0.167,1.42) Sometimes: 0.778 (-0.861,2.417) Rarely: 1.822 (-3.170,6.184)	0.122 0.352 0.474	-0.103 (-0.507,0.302)	0.618	-0.254 (-0.065,0.558)	0.540	Mostly: -0.402 (-1.459,0.654) Sometimes: -0.201 (-2.234,1.831) Rarely: 3.366 (-1.361,8.093)	0.455 0.846 0.163
Confidence in safety (Ref: always)	0.070 (-0.076-0.217)	0.347	0.228 (-0.555,1.011)	0.568	Mostly: 0.098 (-0.775,0.971) Sometimes: -0.083 (-1.963,1.797) Rarely: NA	0.826 0.931 NA	0.054 (-0.113,0.221)	0.552	0.136 (-0.78,1.051)	0.771	Mostly: -0.407 (-1.563,0.75) Sometimes: 1.369 (-0.328,3.067) Rarely: NA	0.491 0.114 NA
Unexpected harm (Ref: no)	-0.114 (-0.340,0.113)	0.323	-	-	Physical Harm: -0.018 (-1.771,1.736) Emotional Distress: 0.417 (-0.683,1.516) **Both: 1.577 (0.486,1.442)**	0.984 0.458 **0.005**	-0.061 (-0.32,0.197)	0.641	-	-	Physical Harm: 0.435 (-1.2, 2.069) Emotional Distress: 0.2 (-1.042,1.442) Both: -0.261 (-1.636,1.113)	0.602 0.753 0.709
Harm discussed (*n*=50)	0.103 (-1.982,2.188)	0.923	-	-	-	-	0.599 (-1.892,3.09)	0.637	-	-	-	-

*AHPEQS *Australian Hospital Patient Experience Question Set, *reg *regression analysis, *Ologit *ordered logistic regression analysis, *Mlogit *multinomial logistic regression analysis, *Ref *reference, *NA *Not analysable; Bolt text indicates *p* value =<0.05

Two FACIT-TS-G items (“*would you recommend this treatment to others*?” 84% and “*would you choose this treatment again?*” 82%), and one AHPEQS item (“*I felt confident in the safety of my treatment and care*” 84%) demonstrated ceiling effect (Table 2). There were no floor effects observed.

The EQ5D-5L responsiveness ES was deemed trivial at 0.16 and 0.15 for all-cause PIVC failure and multiple attempts at PIVC insertion, respectively. The responsiveness statistic demonstrated similar results at 0.16 for both outcomes of interest; the EQ5D-5L SRM overall was 0.157.

## Discussion

Our study successfully examined the usefulness of one generic HRQoL and two patient-reported experience instruments among patients with PIVCs. Several individual items demonstrated usefulness in discriminating the incidence of both multiple PIVC insertion attempts and all-cause PIVC failure, however our investigation suggested the measures may not be useful as a whole. While both EQ5D-5L and FACIT-TS-G have previously been validated (and found reliable) in various other clinical contexts, much of this work has related to complex and/or chronic health conditions (involving multiple human systems) such as multiple-sclerosis (FACIT-TS-G) [32], long-COVID [33], and cancer (FACIT-TS-G; EQ5D-5L) [34, 35]. Therefore, they may not be suitable to detect the nuances of a small (nevertheless important) *element*s (or interventions) of healthcare interactions (a phenomenon previously identified in relation to the EQ5D-5L) [35]. Notably AHPEQS, as a comparatively new instrument has undergone little validation to date [36].

Despite this, all-cause failure was correlated with significant differences in responses to several individual *items*, warranting further investigation. For those with all-cause PIVC failure, participants were more likely to report increased problems with ‘*mobility*’ and ‘*usual activities’* in the EQ5D-5L, however, the overall EQ5D-5L utility score demonstrated trivial responsiveness. Consequently, the correlation between PIVC failure and these two items may be spurious, particularly if correlations of covariates are large (and self-predictive) (e.g., patient acuity and incidence of PIVC failure may increase in a collinear manner) [37].

Overall, the PREMs (AHPEQS and FACIT-TS-G) included seven items with significant results. These items at times aligned with themes identified in qualitative studies of patients’ lived experiences of PIVCs [4, 38]. For example, patients who experienced all-cause failure were more likely to report lower ‘*satisfaction*’ and reported “*both physical and emotional harm*” related to their PIVCs (rather than physical or emotional harm, individually). However, for both FACIT-TS-G and AHPEQS, the direction of effect was reversed for several items, casting doubt on their usefulness. For example, participants with all-cause failure reported *increased* likelihood of reporting doctors helping them “*evaluating the effects of their PIVC”* (FACIT-TS-G) and *higher* involvement in ‘*decision-making*’, in addition to greater ‘*inter-staff communication*’ (AHPEQS). This suggests patients who experienced PIVC failure were more likely to *discuss* this with their treating clinicians; and note increased discussion between staff members related to it.

Participants who experienced multiple PIVC insertion attempts were significantly more likely to report the *side effects* of their PIVC being ‘*a little worse*’ and lower *satisfaction* (on the FACIT-TS-G only). No other significantly significant results were noted. In contrast, multiple insertion attempts (and resulting pain, discomfort, and anxiety) are a common issue of high importance identified in recent qualitative studies [4, 38]. Use of such qualitative data to support psychometric validation is essential when determining construct validity [39]. Thus, largely, all three instruments were inadequate for use in this context.

There were three items (from a total of 16, between the three instruments) which demonstrated ceiling effect, demonstrating a generally high level of variability among most items analysed. Of these, two FACIT-TS-G items were yes/no questions (“*would you recommend this treatment to others”* and “*would you choose this treatment again*”), which suggests they were meaningfully eliciting a response to identify patients with problems.

### Limitations

Limitations of this study include the large attrition of participants between completion of the EQ5D-5L at baseline (time 0, device insertion), and time 1 (device removal, or study completion), however impact of attrition is likely to be low given the similarities of participant and device characteristics, and outcomes of T0 and T1 samples. Whilst the above testing of the performance of the selected instruments is consistent with previously published assessments and is limited by the available data collected alongside a randomised control trial, it is noted that the above analyses do not represent all elements considered in the validation of an instrument (see for example COSMIN). However, whilst including a broader set of performance metrics would provide a more complete understanding of the above instruments’ validity within this context, the limitations and capabilities of these instruments as identified within this restricted set of assessment remain.

Additionally, findings are limited by the use of PIVC-contextualised responses for EQD-5L and FACIT-TS-G, to the exclusion of AHPEQS, for which patients were asked to elicit responses related to their *whole* hospital experience. AHPEQS responses were also limited to one site (Site 1), resulting from timing of parent trial recruitment periods. Furthermore, as this site was limited to two metropolitan hospitals in Queensland, Australia, findings may not be externally generalisable. Despite this, the large number of responses elicited, and the high number of outcomes of interest (PIVC-failure, and multiple insertion attempts) enabled a meaningful analysis. We believe these findings may be useful to clinicians and researchers utilising HRQoL measures and PREMs in a PIVC-specific context in the future.

## Conclusions

Initial investigation of the HRQoL and PREM instruments assessed in this secondary analysis suggest these tools are inadequate in the context of PIVCs among hospitalised patients. Several individual items demonstrated significant results in our analysis, which correlated with similar themes identified in recent qualitative studies Future purpose-built PREM and HRQoL measures, if developed, should consider inclusion of these items, in addition to robust qualitative assessment to ensure their relevance, comprehensiveness and comprehensibility.

### Supplementary Information

**Additional file 1.** Supplementary File 1.

**Additional file 2: Supplementary Table 1.** EQ5D-5L standard regression, ordered logistic regression, multinomial (polytomous) logistic regression results.

**Additional file 3: Supplementary Table 2.** FACIT-TS-G standard regression, ordered logistic regression, multinomial (polytomous) logistic regression results.

**Additional file 4: Supplementary Table 3.** AHPEQS standard regression, ordered logistic regression, multinomial (polytomous) logistic regression results.

## Data Availability

The datasets used and analysed during the current study are available from the corresponding author on reasonable request, and in accordance with original HREC/s restrictions for use.

## Abbreviations

AHPEQS: Australian Hospital Experience Question Set
D: Score Change
EQ5D-5L: EuroQol – Five Dimension – Five Level
ES: Effect Size
FACIT-TS-G: Functional Assessment of Chronic Illness Therapy – Treatment Satisfaction - General
HRQoL: Health Related Quality of Life
PIVC: Peripheral Intravenous Catheter
PREM: Patient Reported Experience Measure
NRS: Numerical Rating Scale
RCT: Randomised Controlled Trial
SD: Standard Deviation
SRM: Standardised Response Mean

## References

[1] Zingg W, Pittet D (2009). Peripheral venous catheters: an under-evaluated problem. Int J Antimicrob.

[2] Marsh N, Webster J, Larsen E, Cooke M, Mihala G, Rickard CM (2018). Observational study of Peripheral Intravenous catheter outcomes in adult hospitalized patients: a multivariable analysis of Peripheral Intravenous catheter failure. J Hosp Med.

[3] Larsen EN, Marsh N, O’Brien C, Monteagle E, Friese C, Rickard CM (2021). Inherent and modifiable risk factors for peripheral venous catheter failure during cancer treatment: a prospective cohort study. Support Care Cancer.

[4] Larsen E, Keogh S, Marsh N, Rickard C (2017). Experiences of peripheral IV insertion in hospital: a qualitative study. Br J Nurs.

[5] Patel CN, Swartz MD, Tomasek JS, Vincent LE, Hallum WE, Holcomb JB (2019). The effects of missed doses of antibiotics on hospitalized patient outcomes. J Surg Res.

[6] Dychter SS, Gold DA, Carson D, Haller M (2012). Intravenous therapy: a review of Complications and economic considerations of peripheral access. J Infus Nurs.

[7] Saliba P, Hornero A, Cuervo G, Grau I, Jimenez E, Berbel D (2018). Interventions to decrease short-term peripheral venous catheter-related bloodstream Infections: impact on incidence and mortality. J Hosp Med.

[8] Rhodes D, Cheng A, McLellan S, Guerra P, Karanfilovska D, Aitchison S (2016). Reducing Staphylococcus aureus bloodstream Infections associated with peripheral intravenous cannulae: successful implementation of a care bundle at a large Australian health service. J Hosp Med.

[9] Cleeland CS, Sloan JA, Group AO (2010). Assessing the symptoms of cancer using patient-reported outcomes (ASCPRO): searching for standards. J Pain Symptom Manage.

[10] Yang LY, Manhas DS, Howard AF, Olson R (2018). Patient-reported outcome use in oncology: a systematic review of the impact on patient-clinician communication. Support Care Cancer.

[11] Weldring T, Smith SM (2013). Article commentary: patient-reported outcomes (pros) and patient-reported outcome measures (PROMs). Health Serv Insights.

[12] de Bienassis K, Kristensen S, Hewlett E, Roe D, Mainz J, Klazinga N (2022). Measuring patient voice matters: setting the scene for patient-reported indicators. Int J Qual Health Care.

[13] Larsen E, Wickins J, Marsh N, Byrnes J, Rickard C. Patient reported outcomes and experiences for peripheral venous catheters: A scoping review. *(In draft)* 10.12968/bjon.2021.30.19.S30 34723667

[14] Australian Hospital Patient Experience Question Set: Australian Commission on Safety and Quality in Health Care. ; 2019 [Available from: https://www.safetyandquality.gov.au/our-work/indicators-measurement-and-reporting/australian-hospital-patient-experience-question-set]. Accessed 16 Dec 2022.

[15] Questionnaires FACITorg. FACIT.org; 2020 [Available from: https://www.facit.org/]. Accessed 16 Dec 2022.

[16] EQ-5D Instruments: EuroQol Research Foundcation. ; 2020 [Available from: https://euroqol.org/eq-5d-instruments/.] Accessed 16 Dec 2022.

[17] Rickard C, Larsen E, Walker R, Mihala G, Byrnes J, Saiyed M et al. Integrated versus nonintegrated peripheral intravenous catheter in hospitalized adults (OPTIMUM): a randomized controlled trial. J Hosp Med. 2022;18(1):21–32. 10.1002/jhm.12995 PMC10099685 36372995

[18] Rickard CM, Marsh N, Webster J, Runnegar N, Larsen E, McGrail MR (2018). Dressings and securements for the prevention of peripheral intravenous catheter failure in adults (SAVE): a pragmatic, randomised controlled, superiority trial. Lancet.

[19] Devlin NJ, Brooks R (2017). EQ-5D and the EuroQol group: past, present and future. Appl Health Econ Health Policy.

[20] Hung M-C, Lu W-S, Chen S-S, Hou W-H, Hsieh C-L, Wang J-D (2015). Validation of the EQ-5D in patients with traumatic limb injury. J Occup Rehabil.

[21] Nowels D, McGloin J, Westfall JM, Holcomb S (2005). Validation of the EQ-5D quality of life instrument in patients after Myocardial Infarction. Qual Life Res.

[22] Norman R, Cronin P, Viney R, King M, Street D, Ratcliffe J (2009). International comparisons in valuing EQ-5D health states: a review and analysis. Value Health.

[23] Viney R, Norman R, King MT, Cronin P, Street DJ, Knox S (2011). Time trade-off derived EQ-5D weights for Australia. Value Health.

[24] Centers for Disease Control and Prevention (CDC). CDC/National Healthcare Safety Network Surveillance definition of healthcare-associated infection and criteria for specific types of infections in the acute care setting. 2022. [Available from: https://www.cdc.gov/nhsn/pdfs/pscmanual/17pscnosinfdef_current.pdf]. Accessed 21 Mar 2023.

[25] Rowen D, Young T, Brazier J, Gaugris S (2012). Comparison of generic, condition-specific, and mapped health state utility values for Multiple Myeloma cancer. Value Health.

[26] Kularatna S, Byrnes J, Chan YK, Ski CF, Carrington M, Thompson D, Stewart S, Scuffham PA (2017). Comparison of the EQ-5D-3L and the SF-6D (SF-12) contemporaneous utility scores in patients with Cardiovascular Disease. Qual Life Res.

[27] Davidson M, Michalos AC (2014). Known-groups validity. Encyclopedia of Quality of Life and Well-Being Research.

[28] Mokkink L, Terwee C, de Vet H (2012). Key concepts in clinical epidemiology: responsiveness, the longitudinal aspect of validity. J Clin Epidemiol.

[29] Walters SJ, Brazier JE (2003). What is the relationship between the minimally important difference and health state utility values? The case of the SF-6D. Health Qual Life Outcomes.

[30] Middel B, Van Sonderen E. Statistical significant change versus relevant or important change in (quasi) experimental design: some conceptual and methodological problems in estimating magnitude of intervention-related change in health services research. Int J Integr Care. 2002;2:e15. 10.5334/ijic.65 PMC1480399 16896390

[31] Mokkink LB, Terwee CB, Knol DL, Stratford PW, Alonso J, Patrick DL (2010). The COSMIN checklist for evaluating the methodological quality of studies on measurement properties: a clarification of its content. BMC Med Res Methodol.

[32] Engebretson E, Seale RA, Valdez B, Vollmer TL, Medina LD (2020). Validation of the functional assessment of chronic Illness therapy–general treatment satisfaction (FACIT-TS-G) in multiple sclerosis. Mult Scler Relat Disord.

[33] Tran V-T, Riveros C, Clepier B, Desvarieux M, Collet C, Yordanov Y (2022). Development and validation of the Long Coronavirus Disease (COVID) Symptom and Impact Tools: a set of patient-reported instruments constructed from patients’ lived experience. Clin Infect Dis.

[34] Peipert JD, Beaumont JL, Bode R, Cella D, Garcia SF, Hahn EA (2014). Development and validation of the functional assessment of chronic Illness therapy treatment satisfaction (FACIT TS) measures. Qual Life Res.

[35] Leunis A, Redekop W, Lowenberg B, Uyl-de Groot C (2012). UT1 the calculation of quality of Life Utilities for Acute Leukemia: a comparison between eq. 5D-5L and QlQ-C30. Value Health.

[36] Nelson HJ, Pienaar C, McKenzie K, Williams AM, Swaminathan G, Mӧrelius E. Development of the Australian hospital patient experience question set for parents. Collegian. 2023;30(2):13–221.

[37] Christensen KB, Comins JD, Krogsgaard MR, Brodersen J, Jensen J, Hansen CF (2021). Psychometric validation of PROM instruments. Scand J Med Sci Sports.

[38] Plohal A (2021). A qualitative study of adult hospitalized patients with difficult venous access experiencing short peripheral catheter insertion in a hospital setting. J Infus Nurs.

[39] Aroian KJ, Schappler-Morris N (1996). Using qualitative data for estimating construct validity of standardized measures. J Nurs Meas.

