# Supplementary material for: Health-related quality of life and experience measures, to assess patients’ experiences of peripheral intravenous catheters: a secondary data analysis
Source: Health Qual Life Outcomes. 2024 Jan 2;22:1. doi: 10.1186/s12955-023-02217-8 (PMC10762939; doi:10.1186/s12955-023-02217-8)
Supplement: Supplementary file 1 — Additional file 1. Supplementary File 1. [file 12955_2023_2217_MOESM1_ESM.docx]

**Supplementary File 1**

**Introduction statements:**

#### EQ5D-5L

The introduction statement during the conduct of the RCT was as follows: “*You may remember from our conversation when I/we invited you to participate in this study, that I/we was/were going to come back to ask you a few questions about your experience with your IV catheter. I/we need to ask you six questions. It will take us about five minutes to go through them. I will read each question and the possible answers to you, so you can select the answer that best reflects your experience with your IV catheter. If you are unsure, that's OK, we can skip that one and continue with the next*.”

#### FACIT

The introduction statement was as follows: “*You may remember from our conversation when I/we invited you to participate in this study, that I/we was going to come back to ask you a few questions about your experience with your IV catheter. I/we need to ask you eight questions. It will take us about five minutes to go through them. I will read each question and the possible answers to you, so you can select the answer that best reflects your experience with your IV catheter. If you are unsure, that's OK, we can skip that one and continue with the next. Please evaluate your experience with your IV catheter. If you have not completed your treatment, please answer the questions the best you can. All of your answers will be kept confidential*”.

#### AHPEQS

The introduction statement was as follows: “*You may remember from our conversation when I/we invited you to participate in this study, that I/we was going to come back to ask you a few questions about your experience in the hospital. I/we need to ask you 12 questions. It will take us about five minutes to go through them. I will read each question and the possible answers to you, so you can select the answer that best reflects your hospital experience. If you are unsure, that's OK, we can skip that one and continue with the next*.”
